# Potential risk analysis and experience summarization of unstable factors of cranial fixation devices in neurosurgical operations: three-case reports and systematic review

**DOI:** 10.1186/s41016-021-00244-2

**Published:** 2021-04-28

**Authors:** Gaopeng Cheng, Shuyu Hao, Zhifen Ye, Bao Wang, Bin Huangpu, Pengfei Zhang, Hao Wang, Qiang Hao

**Affiliations:** 1 Department of Neurosurgery, Heping Hospital Affiliated to Changzhi Medical College, Changzhi, Shanxi Province China; 2 Department of Neurosurgery, Beijing Tiantan Hospital, Capital Medical University, Beijing, China; 3 Department of Neurosurgery, Jinzhong First People’s Hospital, Jinzhong, Shanxi Province China; 4 Department of Neurosurgery, Linfen People’s Hospital, Linfen, Shanxi Province China; 5 Department of Neurosurgery, The Seventh Medical Center of Chinese People’s Liberation Army General Hospital, Beijing, China

**Keywords:** Cranial fixation devices, Neurosurgical operations, Unstable factors, Potential risk, Experience summarization

## Abstract

**Background:**

The use of cranial fixation devices in neurosurgery is very common, which is considered to be an important auxiliary method for many craniotomies. However, previous studies have reported complications of using cranial fixation devices, including brain tissue, nerve and blood vessel damage, scalp laceration, subcutaneous hematoma, etc. Some of the complications are serious and even potentially fatal, and the causes of which may be related to the incorrect use of cranial fixation devices. Although there are no serious complications in our review, the cause of that needs to be further summarized and analyzed, as so to minimize the serious consequences caused by the cranial fixation device slippage and ensure the safety of the patients’ surgical procedure.

**Case presentation:**

In our recent work, we have continuously found three cases of unstable cranial fixation devices, which make us to analyze the possible factors and summarize experience combined with the review of other senior neurosurgeons (more than 3 years of working experience) from different departments of neurosurgery.

**Conclusions:**

Based on our recent incidents of unstable cranial fixation and the experience of investigating and analyzing senior doctors from different neurosurgery centers, we summarized experience to minimize the risk of unstable cranial fixation. We tried a variety of options, including a safe anatomical location for cranial fixation, teamwork, and communication with anesthesiologists and itinerant nurses, to ensure the stability of the patient’s cranial fixation devices. The data obtained in this survey has great limitations, including the doctor’s personal prejudice and dependence on anecdotal memories. Therefore, the data should be interpreted with caution. However, there are still some modes that can help to better understand the use of safe cranial fixation. Based on the above research and analysis, we have made recommendations that may help neurosurgeons to avoid preventable complications

## Background

The use of cranial fixation devices in neurosurgery is very common and considered to be an important auxiliary method for many craniotomies. Gardner designed the first cranial fixation devices for sitting surgery in 1935, and later Mayfield cranial fixation devices were developed to locate the skull and prevent movement in the proceed of neurosurgery as a safe equipment [1, 2].

However, previous studies have reported complications of using cranial fixation devices in adults and children [3–5]. Some of the complications are serious and even potentially fatal, and the causes of which may be related to the incorrect use of cranial fixation devices [4, 5]. Among many adverse events, injuries caused by falling of cranial fixation devices are often the most serious and may be life-threatening.

Despite few cases published, several neurosurgeons discussed complications of cranial fixation devices during their careers in informal conversations, suggesting that their risks must be underestimated [3]. In our recent work, we have continuously found three cases of unstable cranial fixation devices, which reminded us to analyze the possible factors and summarize experience combined with the review of other senior neurosurgeons from different departments of neurosurgery.

## Case presentation

Case 1, male, 53 years old, was diagnosed with cerebellar vermin meningioma. The patient was adopted left abdominal supine position and fixed with Mayfield head frame, where double pins were located in the left temporal part and a single pin was located in the right temporal part. The pressure of pins achieved 70 pounds. The surgical approach adopted the posterior median approach, and 2 holes were drilled during the operation. The whole operation time was 4 h. The patient showed head movement when we sutured scalps. When the Mayfield head frame was removed after the operation, the pressure of pins was changed into 50 pounds.

Case 2, female, 42 years old, was diagnosed with acoustic neuroma located in the left cerebellopontine angle (CPA). The patient was adopted right abdominal decubitus and fixed with Mayfield head frame, where double pins were located in the left temporal part and a single pin was located in the right temporal part. The pressure is 60 pounds. The surgical approach adopted the right retro-sigmoid approach, and 2 holes were drilled during the operation. The whole operation time was 4 h. The patient showed head movement when we sutured scalps. When the Mayfield head frame was removed after the operation, the pressure of pins was changed into 40 pounds.

Case 3, female, 61 years old, was diagnosed with meningioma located in the left frontraoparietal. The patient was adopted supine position and fixed with Mayfield head frame, where double pins were located in the right temporal part and a single pin was located in the left temporal part. The pressure is 60 pounds. The surgical approach adopted the left frontraoparietal approach, and 2 holes were drilled during the operation, and the whole operation time was 4 h. When the head frame was removed after the operation, the head frame scale was found to be 50 pounds.

**Fig. 1:**
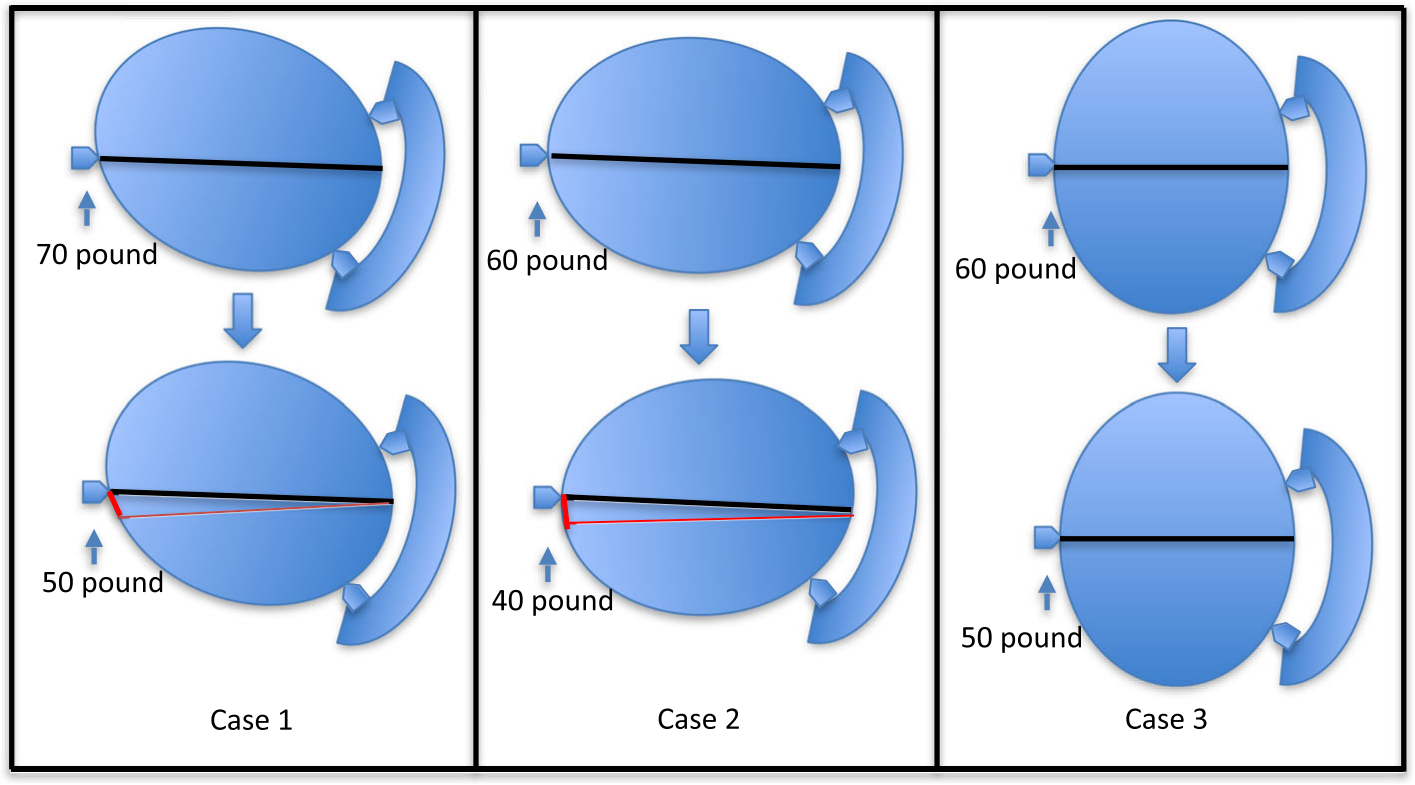
Illustration figures for three cases with unstable cranial fixation devices

These incidents aroused our great attention. If we did not discover and summarize the experience in time, it may appear again in the subsequent work and cause serious consequences. To this end, we also communicated with senior physicians and investigated cerebral fixation device instability events that occurred among different neurosurgeons. All illustration figures of the three cases are shown in Fig. 1.

## A survey of senior doctors

We investigated the neurosurgeons from Beijing Tiantan Hospital, Jinzhong First People’s Hospital, Linfen People’s Hospital, and The Seventh Medical Center Of Chinese People’s Liberation Army General Hospital, respectively. The content of this survey contains operation position, emergency operation, depth of anesthesia, pressure of pins, the skin of the head, time of operation, and appearance of unstabilization.

## Discussion

Among the seven doctors surveyed, five doctors were found to have experienced cranial fixation device instability events, of which one doctor experienced one incidence, three doctors experienced two incidences, and one doctor experienced three incidences. In all incidences, there is no complete slip of head frame and serious complications, and the main results are partially displacement of cranial fixation devices and reduction of pressure. In addition, with the growth of these senior doctors, the incidence of these events has declined significantly, and most of the incidents occurred during the period of serving as a young doctor.

**Fig. 2:**
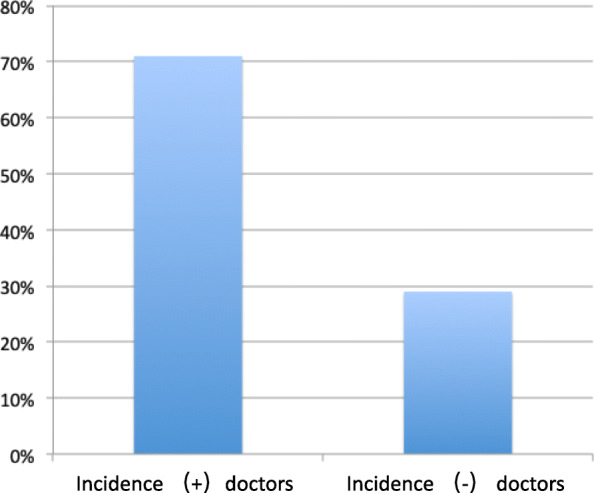
Rate of doctors with cranial fixation incidents. Among the doctors surveyed, about 71% doctors experienced unstable incidences of cranial fixation devices

In the events of cranial fixation device instability, the ratio of men and women is the same. Among these incidents, three patients occurred in the supine position, accounting for 30%, and seven patients occurred in the lateral/prone position, accounting for 70% (Fig. 1). Among the ten incidents, four incidents of them occurred in emergency surgery, and six incidents were routine surgery. Four incidents had involuntary movements before removing cerebral fixation devices. The pressure of cranial fixation pins is strictly 60 pounds for women and 70 pounds for men. Among them, four patients are with thicker soft tissues in the head, and six patients with normal and thin skins. In the events of unstable cranial fixation devices, there were no serious complications. Among them, four patients showed head pin displacement, and the distance was less than 1 cm; six patients showed pressure reduction to 40–50 pounds with no obvious tack displacement. Although there are no serious complications, the cause of that needs to be further summarized and analyzed, as so to minimize the serious consequences caused by the cranial fixation device slippage and ensure the safety of the patients’ surgical procedure. Among the doctors surveyed, about 71% doctors experienced unstable incidences of cranial cerebral fixation devices (Fig. 2).

Complications of cranial fixation devices have been reported in the literature, including skull fractures, needle infections, venous air embolism, and subdural and epidural hematoma [6, 7]. Despite owning these potential risks, cranial fixation devices provide the unique advantages of fixation and stability in cranial operations, especially for high-precision surgery, requiring that the head frame installation must be strictly fixed. At the same time, cranial fixation device fixation can be used to prevent concomitant pressure ulcers and eye complications [8, 9]. Based on the survey of multiple senior doctors in different hospitals, we investigate the stability of the cranial fixation device installation, and the possible potential risks are summarized.

From these results, we found that patients in the lateral prone position were more prone to induce cranial fixation device instability, indicating that there were large instability factors in the prone/lateral prone abdominal position, which may be due to the weight of bodies was more distributed on the cranial fixation devices than the supine position. It required that we should pay more special attention to the stability of the cranial fixation device installation when patients were performed in the prone/lateral prone abdominal position. Among the ten patients, 40% of them are emergency surgery and 60% are routine surgery. The proportion of routine surgery seems to be higher than emergency surgery; however, the overall number of routine surgery is much higher than that of emergency surgery. From this point, we can obtain that the probability of cranial fixation device instability in emergency surgery is much higher than the proportion of routine surgery. These results showed that the preparation for emergency surgery might not be as full as in routine surgery, and errors were more likely to occur.

In addition, 40% patients had involuntary movements before removing the cranial fixation devices. And in one patient who recently had unstable cranial fixation devices, we paid special attention to the installation position and the amount of pressure applied after installing the headgear and checked with the superior doctors and traveling nurse. At the end of the operation, we found that the patient’s head appeared involuntary activities. We communicated with the anesthesiologist in me to enhance the control of anesthetic drugs. In the end, we found that the pressure of the cranial fixation devices was changed from 70 pounds to 50 pounds. Two of the three patients who recently found the cranial fixation device unstable were accompanied by involuntary shaking of the head during the operation. These results suggested that the depth of patient’s anesthesia also plays an important role in the stability of cranial fixation devices. The shaking of the patient’s head during the operation may affect the stability of the cranial fixation devices.

Among all patients, the pressure of cranial fixation devices is strictly controlled for women 60 pounds and men for 70 pounds. Among them, there were four cases of unstable cranial fixation devices in patients with thicker tissues, accounting for 40%, and six cases of normal and thin tissue patients accounted for 60%. These results indicated that in patients with a thicker scalp, the pressure should be appropriately increased to prevent the head pins to shift or even slip. The length of the operation time is also closely related to the stability of the tissues. With the extension of the operation time, the higher proportion of cranial fixation device slipping or displacement happens.

In the cases investigated, there were no serious complications. Four cases showed cranial fixation device displacement, and the distance was less than 1 cm. Another six patients showed pressure reduction to 40–50 pounds, and no obvious head displacement occurred. Although there were no serious complications, the cause of its occurrence needs to be further summarized and analyzed, so as to minimize the serious consequences and ensure the safety of patient’s surgical procedure.

**Fig. 3:**
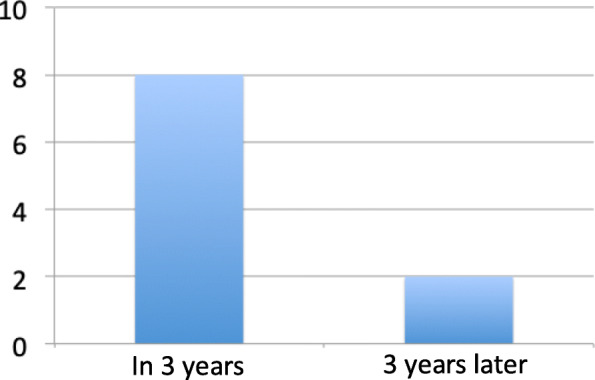
Working years of doctors with cranial fixation incidents. About 80% occurred in the first 3 years of their work career, rarely in the subsequent work period

Among the doctors surveyed, about 71% doctors experienced unstable incidences of cranial fixation devices, and 80% occurred in the first 3 years of their work career, rarely in the subsequent work period. This showed that the stability of the cranial fixation devices in the beginning of the work was closely related to the doctor’s experience (Fig. 3 and Table 1). This was also related to the communication between the anesthesiologist and the traveling nurse through the operation.

**Table 1:**
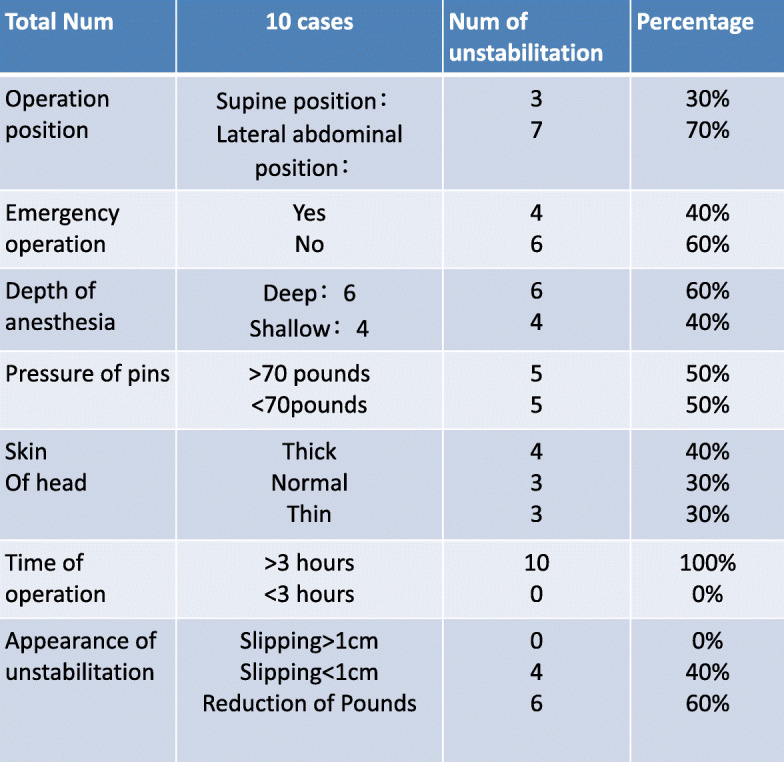
Summarization of unstable factors of cranial fixation devices in neurosurgical operations

Based on our recent unstable cranial fixation incidents and the experience of investigating and analyzing senior doctors from different neurosurgery centers, we conducted experience analysis and summary to minimize the risk of unstable cranial fixation pins. We tried a variety of options, including a safe anatomical location for cranial fixation, teamwork, and communication with anesthesiologists and itinerant nurses to ensure the stability of the patient’s cranial fixation devices. Although the statistical value of these 10 cases is limited, just based on empirical analysis and retrospective studies, more clinical studies can be conducted to assess related risks and make recommendations for the safe installation of the cranial fixation devices.

## Conclusions

The data obtained in this survey has great limitations, including the doctor’s personal prejudice and dependence on anecdotal memories. Therefore, the data should be interpreted with caution. Despite these limitations, there are still some modes that can help to better understand the use of safe cranial fixation. Based on the above research and analysis, we have made recommendations that may help avoid preventable complications.

**Fig. 4:**
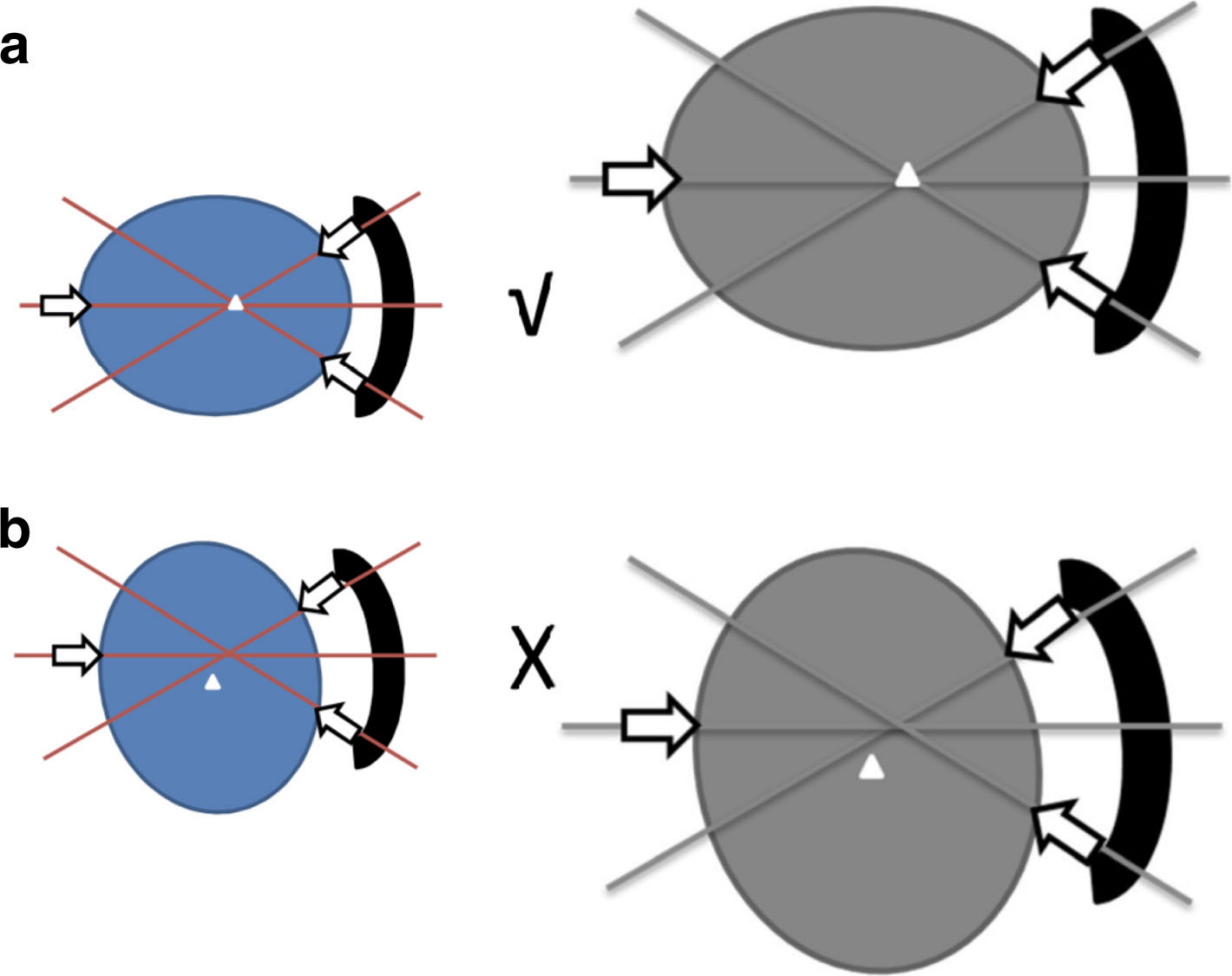
The relation between the position of the cranial fixation devices and the center of the head. **a** The center of the head is located at the upper part of the three-point center of gravity of the head frame. **b** The center of the head is located at the lower part of the three-point center of gravity of the head frame

**Fig. 5:**
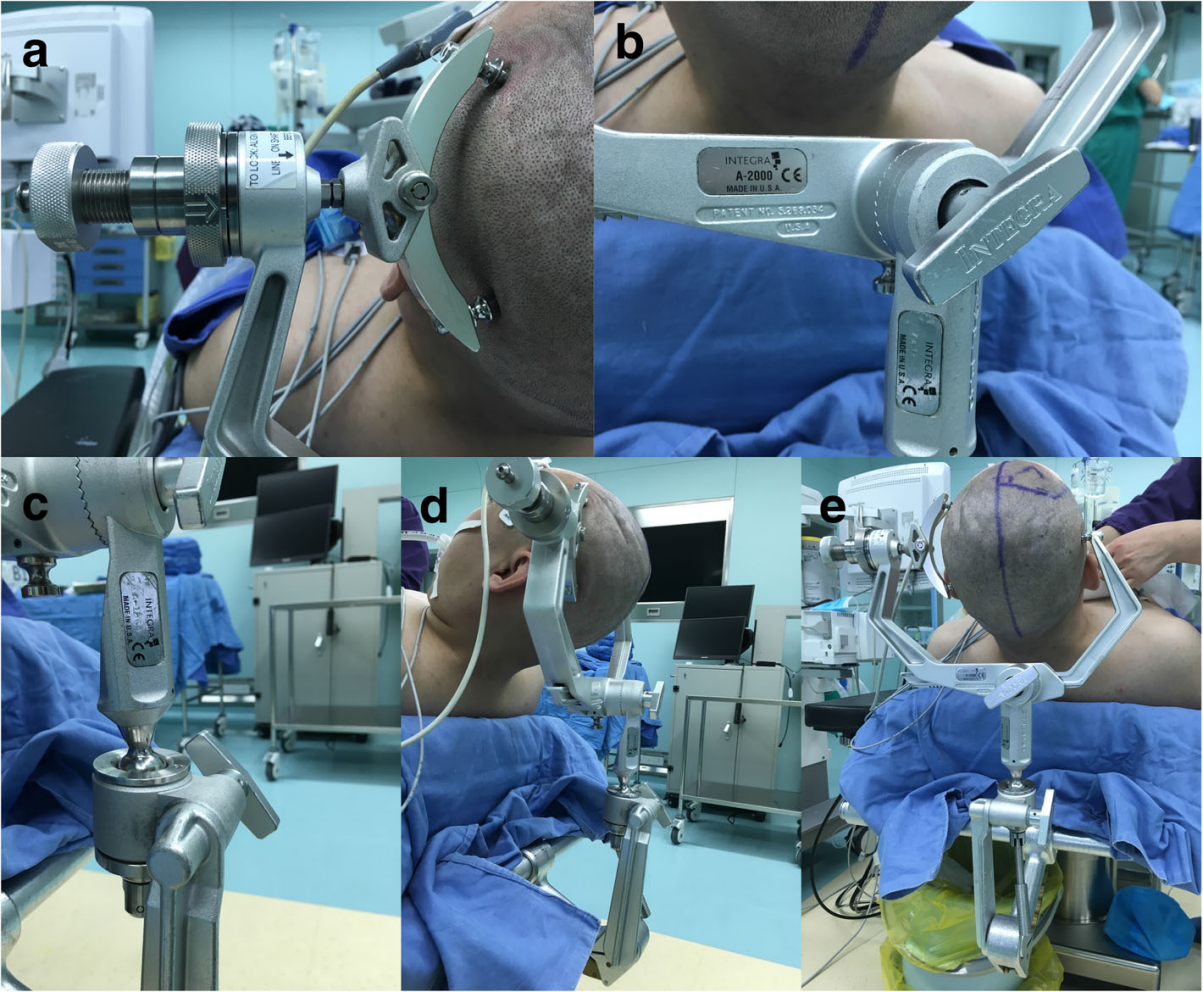
The standard positions of the cranial fixation devices. **a** Two pins located up and down position of fixes position balanced. **b** The head frame is tightly connected with the fixing device. **c** The cardan shaft keeps vertical. **d** Side view of the standard position of cranial fixation devices. **e** Positive view of the standard position of cranial fixation devices

The following suggestions are based on the survey responses and our own practical experience (Figs. 4 and 5):

1. Strictly follow the process to install cranial fixation devices, and at least two doctors are present to verify the stability of cranial fixation devices. The junior doctors need to install the head frame with the assistance of the senior doctors, and gradually accumulate experience;
2. Fully assess the patient’s head circumference size, weight, and skin thickness. In patients with a heavier head and thicker scalp, we should increase the pressure appropriately to ensure the stability of the cranial fixation devices;
3. The position of the cranial fixation devices needs to be carefully analyzed to ensure that the center of the head is located at the upper part of the three-point center of gravity of the head frame, so that it is possible to ensure that the cranial fixation devices support the head effectively;
4. In some complex surgical positions, more attention should be paid to the stability of the cranial fixation devices. Due to various changes in its body angle, there are many variable influencing factors;
5. The prolonged operation time may increase the factors of cranial fixation device instability. In patients with long surgical events, the stability of the cranial fixation devices can be checked intraoperatively;
6. During the operation, try to avoid unnecessary pressure on the patient’s head to cause the instability of the head nail;
7. Fully communicate with the anesthesiologist to determine the end time of the operation, so that the anesthesiologist can better evaluate the use of the anesthetic drugs;
8. The re-check with the itinerant nurse is an indispensable part. In our recent incidents, it is that the itinerant nurse discovered and rigorously verified consequent operations that the discussion and empirical analysis of this article.

## Data Availability

Data used to support the findings of this study are included within the article.

## References

[1] Baerts WD, De Lange JJ, Booij LH, Broere G (1984). Complications of the Mayfield skull clamp. Anesthesiology..

[2] Cabezudo JM, Gilsanz F, Vaquero J, Areitio E, Martinez R (1981). Air embolism from wounds from a pin-type head-holder as a complication of posterior fossa surgery in the sitting position. J Neurosurg.

[3] Grinberg F, Slaughter TF, McGrath BJ (1995). Probable venous air embolism associated with removal of the Mayfield skull clamp. Anesth Analg.

[4] Sade B, Mohr G (2005). Depressed skull fracture and epidural haematoma: an unusual post-operative complication of pin headrest in an adult. Acta Neurochir (Wien).

[5] Yan HJ (2007). Epidural hematoma following use of a three-point skull clamp. J Clin Neurosci.

[6] Aoki N, Sakai T (1989). Modified application of three-point skull clamp for infants. Neurosurgery.

[7] Lee M, Rezai AR, Chou J (1994). Depressed skull fractures in children secondary to skull clamp fixation devices. Pediatr Neurosurg.

[8] Berry C, Sandberg DI, Hoh DJ, Mark D, McComb G (2008). Use of cranial fixation pins in pediatric neurosurgery. Neurosurgery.

[9] Poli JC, Zoia C, Lattanzi D, Balbi S (2013). Epidural haematoma by Mayfield head-holder®: case report and review of literature. J Pediatr Sci.

